# Supplementary material for: Fargesin Inhibits EGF-Induced Cell Transformation and Colon Cancer Cell Growth by Suppression of CDK2/Cyclin E Signaling Pathway
Source: Int J Mol Sci. 2021 Feb 19;22(4):2073. doi: 10.3390/ijms22042073 (PMC7922630; doi:10.3390/ijms22042073)
Supplement: Supplementary file 1 [file ijms-22-02073-s001.pdf]

International Journal of *Molecular Sciences*

[Supplementary Information]

Fargesin Inhibits EGF-Induced Cell Transformation and Colon Cancer Cell Growth by Suppression of CDK2/Cyclin E Signaling Pathway

Ga-Eun Lee [1], Cheol-Jung Lee [1,5], Hyun-Jung An [1], Han Chang Kang [1], Hye Suk Lee [1], Joo Young Lee [1], Sei-Ryang Oh [2], Sung-Jun Cho [3], Dae Joon Kim [4] and Yong-Yeon Cho [1,*]

1. Five Figure legends

Supplementary Figure legends

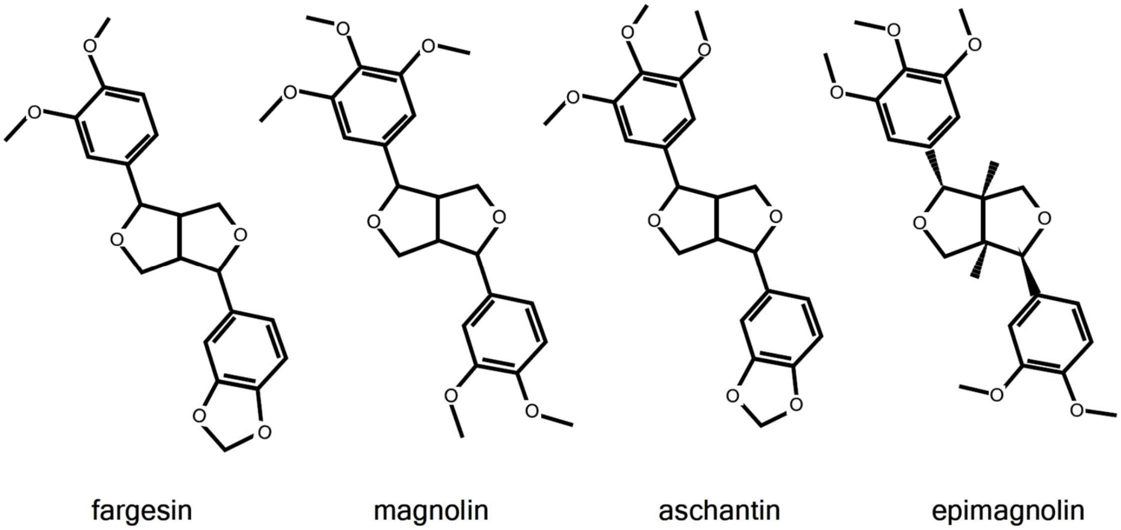

S. Figure S1. Chemical structures of lignans including fargesin, magnolin, aschantin, and epimagnolin.

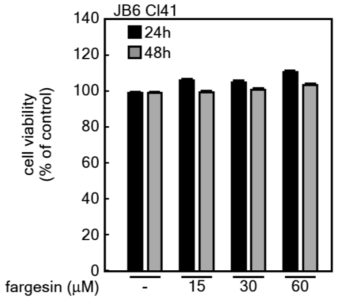

S. Figure S2. Cytotoxicity of fargesin. JB6 Cl41 cells (1×10^4) were seeded into 96-well plates and cultured overnight. The cells were treated with indicated doses of fargesin. The cell toxicities of fargesin were measured by cell viability assay using MTS assay kit at 24 h and 48 h. Data; a triplicate experiment, values; ± SEM, significance; versus non-fargesin-treated control by Student's *t*-test.

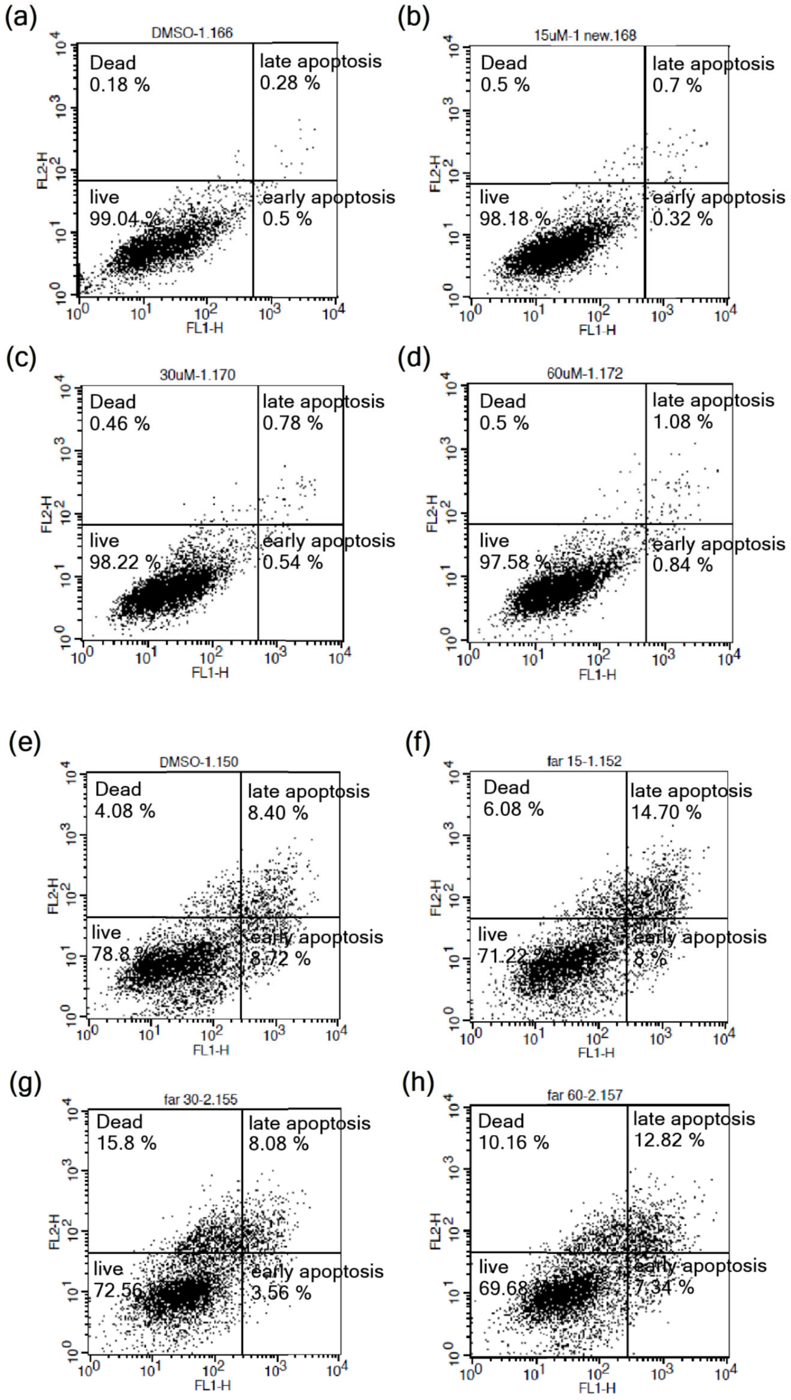

S. Figure S3. Histograms of apoptosis. JB6 Cl41 cells (1 × 10^5) (**a**-**d**), and HaCaT cells (3 × 10^4) (**e**-**h**) were plated into 60-mm culture dishes, and cultured overnight. To examine apoptosis presence, the cells were treated with indicated doses (**a** and **e**: non-treated, **b** and **f**: 15 μM, **c** and **g**: 30 μM, and **d** and **h**: 60 μM) of fargesin in complete cell culture medium for 48 h in JB6 Cl41 cells and for 72 h in HaCaT cells. To explore the effect of fargesin on apoptosis, the flout and attached cells were harvested, fixed with ice-cold 70% ethanol, and then stained propidium iodide. The cell cycle distribution analysis was subjected by flow cytometry using the FACSCalibur flow cytometer. Data were analyzed using ModFit LT.

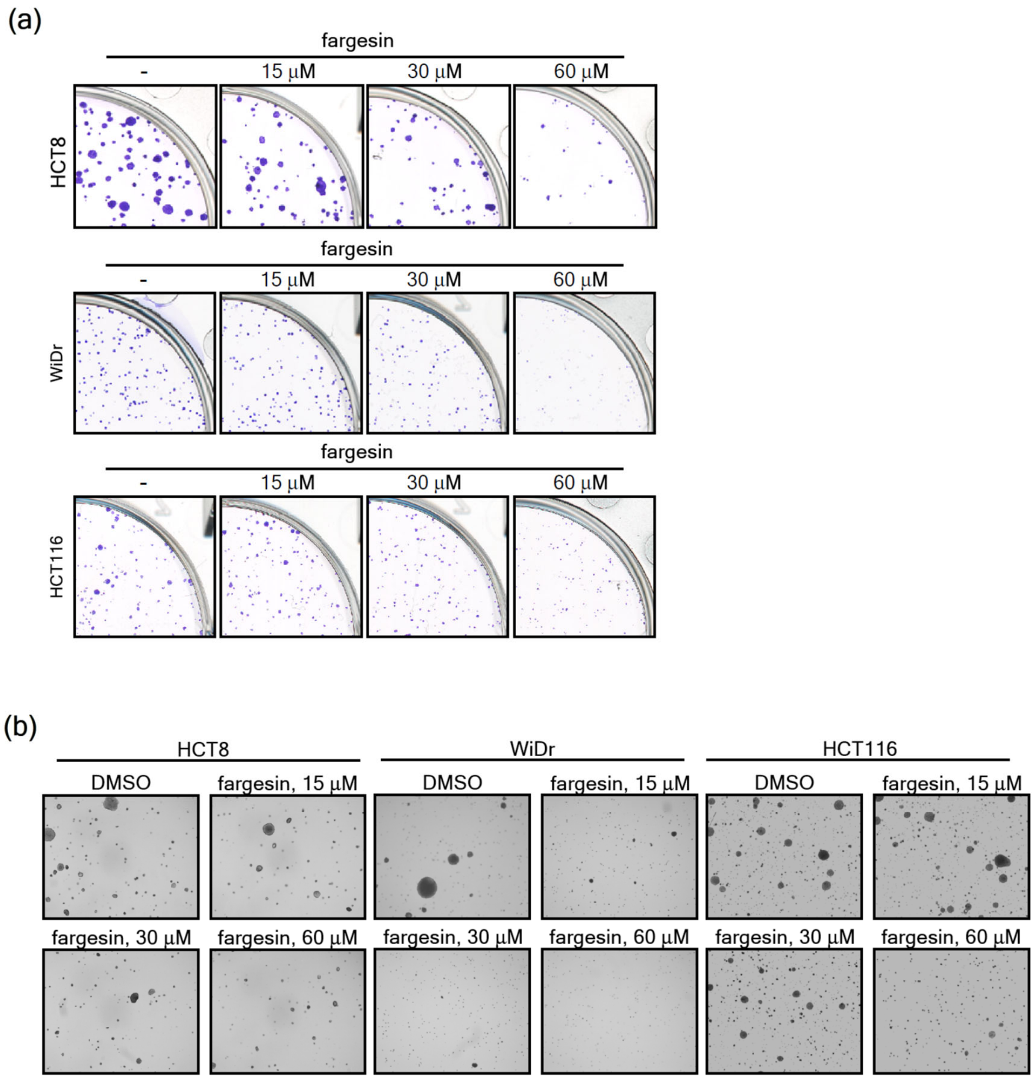

S. Figure S4. Effects of fargesin on the colony growth of colon cancer cells. (**a**) Colon cancer cells including HCT8 (1 × 10^3), WiDr (1 × 10^3), and HCT116 (1 × 10^3) cells were seeded into 6-well plates and incubated overnight. The cells were continuously cultured for 7-10 days. The foci were visualized by Crystal Violet staining (0.05% w/v Crystal Violet, 1% formaldehyde, 1× PBS, 1% methanol) followed by destaining. The formed foci were scanned with an EPSON scanner and scored using Image J software. (**b**) Colon cancer cells including HCT8 (8 × 10^3), WiDr (8 × 10^3), and HCT116 (8 × 10^3) cells suspended in appropriate complete culture medium containing 10% FBS were added to 0.3% agar with indicated doses of fargesin. The cells were cultured for 10-14 days, and the cell colonies were observed under an ECLIPSE Ti inverted microscope. The colony numbers were scored using the NIS-Elements AR (V. 4.0) computer software program, and statistical data of scored colonies are presented at **Figure 2d**.

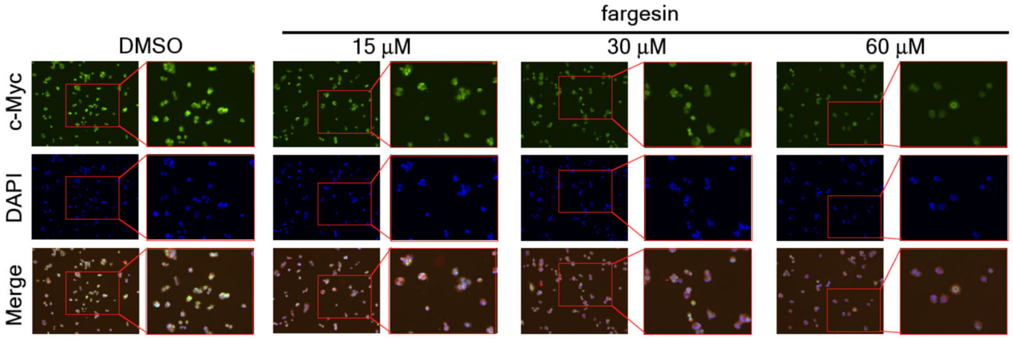

S. Figure S5. Fargesin suppresses c-Myc protein levels in both cytosol and nucleus. WiDr (2×10^4) cells were seeded into 4-chamber slides, cultured overnight, and treated with the indicated doses of fargesin for 6 h. The cells were fixed, permeabilized, and hybridized with c-Myc specific primary antibody and Alexa-488-conjugated secondary antibody. The c-Myc was observed under confocal microscope (×200). The boxed areas of Alexa-488 representing c-Myc are in Figure 5g in main text. The fluorescence intensity from obtained whole photographs was measured by Image J computer program and normalized with DAPI intensity. The statistical analysis data of the fluorescence intensity were presented in Figure 5g (*graphs*) in main text. Green, c-Myc-Alex-488; Blue, nuclei-DAPI. Representative photographs were obtained from three independent experiment.
